# A formative appraisal of female genital schistosomiasis (FGS) score card results against point-of-care gynaecological and molecular parasitological information within four counties of Liberia

**DOI:** 10.1017/S0031182025101133

**Published:** 2025-12

**Authors:** Ayesha E.R. Bell-Gam Woto, Lucas J. Cunningham, Anthony K. Bettee, Harley Seward, Andrew Samorlu, Manfred Yarkpawolo, C. Simeon S. Borbor, T. Henry Kohar, Abedenego S. Wright, Joseph Y. Flomo, Rebecca Vesselee, Tenneh Freeman, Massa Dukuly, Hawa Kormassa Johnson, Farmah Shellie, Chester Peters, Johnathan S. Beglar, Robert Y. Nyumah, Alexander Korpu, Peter Edesiri Ohwoka, Marion Risse, Alexandra Juhasz, E. James La Course, Bernice Dahn, J. Russell Stothard

**Affiliations:** 1 Department of Tropical Disease Biology, Liverpool School of Tropical Medicine, Liverpool, UK; 2 Department of Biological Sciences, TJR Faulkner College of Science and Technology, University of Liberia, Fendall Campus, Monrovia, Liberia; 3 Ministry of Health, Republic of Liberia, Congo Town, Monrovia, Liberia; 4 Department of Environmental Systems Sciences, ETH Zürich, Zürich, Switzerland

**Keywords:** molecular diagnostics, primary health care, *Schistosoma haematobium*, sexual and reproductive health, urogenital schistosomiasis

## Abstract

Liberia (West Africa) has an extensive (co)burden of urogenital and intestinal schistosomiasis; each largely restricted to more inland areas. Where urogenital schistosomiasis is endemic, as both disease surveillance and case management are nascent, many women may unknowingly be living with Female Genital Schistosomiasis (FGS). Using a recently developed FGS score card, we appraised FGS score card valuations with point-of-care gynaecological and molecular parasitological evaluations as undertaken within typical primary care settings of four Liberian counties. A total of 400 women, 100 participants from each of four endemic inland counties, underwent a cursory gynaecological examination using a speculum for visible FGS lesions, undertaken by a midwife, and provided a urine sample that was examined by centrifugation with microscopy for Schistosoma ova. Urine-sediments in ethanol were later analysed with a high-resolution melt (HRM) real-time (rt) PCR assay to screen for Schistosoma genetic markers. Using a combination of clinical and parasitological information, overall prevalence of UGS and FGS was <10% and a single case of putative FGS-associated co-infection with Schistosoma mansoni was observed. Participant interviews with the FGS score cards provided an insight into at-risk lifestyle and environmental factors, e.g. women who fished regularly were more likely to present with FGS whereas those who lived > 15 km from a local river were less likely to present with FGS. In this resource-poor setting of Liberia, active surveillance for FGS with either clinical or parasitological methods remains challenging such that sole future use of the FGS score card is most pragmatic for primary care.

## Graphical abstract

**Figure:**
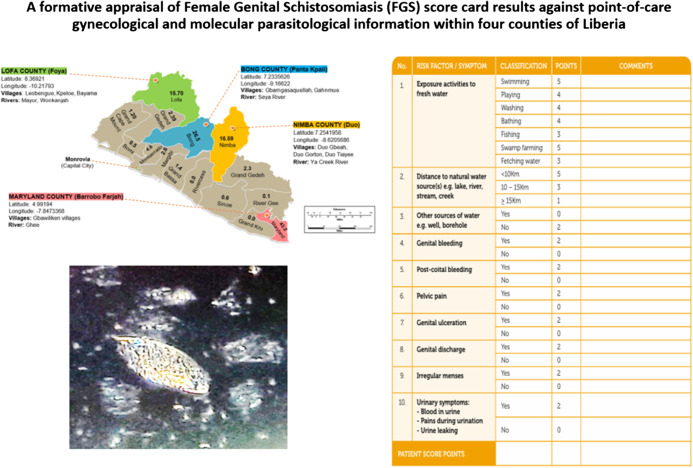

## Introduction

Female genital schistosomiasis (FGS) is a sex and gender-specific manifestation of urogenital schistosomiasis (UGS), affecting several tens of millions of African women who largely suffer unknowingly (Bustinduy et al. 2022; Buonfrate et al. 2025; Mberu et al. 2025). Given its widespread occurrence, FGS is now considered the most common but still cryptic gynaecological problem across sub-Saharan Africa (SSA) (Hotez et al. 2019). Although FGS is largely caused by chronic infection with *Schistosoma haematobium,* other species such as *Schistosoma mansoni*, and more recently *S. haematobium* group hybrids, have now been implicated (Hotez et al. 2019; Sturt et al. 2020; Stothard et al. 2025). With increasing global attention on the importance and interactions between FGS and HIV infection (UNAIDS, 2019), which the UNAIDS Global Strategy 2021–2026 now formally recognizes, a recent collection of papers in *Frontiers in Tropical Disease* shed further light on raising the awareness of FGS across SSA; a research portfolio that encouraged attempts to deliver sustainable actions, for example, better surveillance to guide more tailored health interventions within primary health care facilities. However, several SSA countries have no satisfactory FGS surveillance and case management protocols with an inadequate public health response, despite a substantive burden of UGS (Mberu et al. 2025).

A good example is the West African country of Liberia which has a substantive burden of UGS, as well as intestinal schistosomiasis, each being found in more inland areas where both forms of schistosomiasis can be co-endemic. Given the substantive burden of UGS and following from UK_AID support between 2016 and 2022, guided through the COU**NTD**OWN implementation research programme, attempts were made to kick-start preventive chemotherapy campaigns and establish initial actions on FGS across the health system in Liberia (Nganda et al. 2023). The latter included development and implementation of an FGS score card (see https://countdown.lstmed.ac.uk/sites/default/files/centre/Countdown%20FGS%20Training%20Guide%20Liberia%20-%20Proof%203-%20watermarked.pdf) that involved a rapid community dialogue and assessment which would have otherwise remained invisible within the current resource-poor setting. This was particularly poignant since Liberia was in post-conflict recovery, having an unfortunate history of gender-based violence, and the Ministry of Health was being assisted to best respond to the most pressing issues within its control of neglected tropical diseases (NTDs) nationally. During this period, it also coincided with another debilitating health system shock, controlling the EBOLA outbreak, with limited domestic resources (Thomas et al. 2017).

In brief, Liberia’s devastating civil war, which persisted for 14 years from 1989 to 2003, left the country’s society, systems and infrastructure broken in every sector, including the health system, with only 701 functional health facilities currently serving the country’s population of 5·6 million (Kesselly et al. 2023). Liberia has a core health worker density of 12·8 per 10 000 people, while the WHO has recommended a density target for Liberia of 44·5 health workers per 10 000 population (Ministry of Health, Government of Liberia [GOL], 2018) (LIGIS and ICF International, Liberia Demographic and Health Survey, 2013). According to UNICEF, 1 out of every 10 women die during pregnancy in Liberia (UNICEF Liberia, 2024) with the country known to have one of the highest maternal mortality rates in the world for women dying in childbirth due to poor reproductive diagnostics, as well as one of the highest neonatal mortality rates at 37 deaths per 1000 live births (Case Study 13, WHO, 2022). It is estimated that 11 000 children die annually under the age of 5 due to untreated common childhood illnesses (Liberia Institute of Statistics and Geo-Information Services [LISGIS], 2014) (UNICEF Liberia, 2024). As FGS is the genital manifestation/clinical form of UGS, our study’s primary aim was to conduct a formative appraisal of FGS sore card results across 4 counties highly endemic for UGS. Although unlike UGS, FGS cannot be confirmed solely by the presence of *S. haematobium*, provisional clinical and parasitological assessments of UGS played an important role in assessing possible FGS cases. Our secondary aim was to help and encourage better healthcare delivery for Liberian women with more tailored evidence-based policies by providing recent epidemiological information to support the revised national plan for control of NTDs in Liberia.

## Methods

### Selection of study counties, districts and villages

Epidemiological data from a nationwide country mapping study inspecting primary school children, aged 5–14, with parasitological examination of urine and stool samples were used to select priority endemic counties. This seminal survey was conducted by the Neglected Tropical Diseases Unit at the Liberian Ministry of Health in 2012–2013. From the cartography of these data, the counties of Lofa, Bong and Nimba were selected, whereas the inland portion of Maryland was included as the FGS score card was originally pilot tested there in an initial community assessment and implementation.

**Figure 1.**
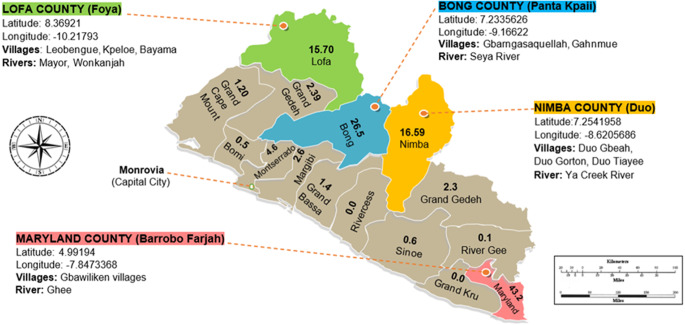
Schematic map of Liberia showing percentage prevalence of UGS within school children from our study-selected counties: Maryland (43·2%), Bong (26·5%), Nimba (16·6%) and Lofa (15·7%). A representative global positioning system (GPS) coordinate for the study villages within each county shown.

The study was conducted in Lofa (Foya District), Bong (Panta Kpaii District), Nimba (Duo District) and Maryland (Barrobo Farjah District) (Figure 1). Each area has distinct socio-cultural and economic contexts, requiring locally tailored community engagement. Lofa County (Foya District), inhabited predominantly by the Kissi tribe, borders Sierra Leone (west) and Guinea (north). Key villages include Leobengu, Kpeloe and Bayama. Rivers, Mayor and Wonkajah are the main water sources. Foya district has 20 569 of Lofa’s 367 376 residents. Bong County (Panta Kpaii District), home mainly to the Kpelle tribe, includes Gbarngasaquella and Ghanmue villages near the Guinea border and N’Zérékoré. N’Zérékoré is Guinea’s second largest city, it was where the 2013–2016 EBOLA virus epidemic started and later reoccurred in 2021 (Raab et al. 2021). Villagers from these sites use the Seya River as their main water source. Although the exact population of these villages are not known, Bong County in general has a population of 467 561 (city population Liberia 2025). Nimba County (Duo District) villages Duo Gbeah, Duo Gorton and Duo Tiayee, inhabited by the Gio and Mano tribes, are about 100 km from the Guinea border. The Ya Creek River is their main water source. Nimba County is the most populous with 621 841 inhabitants (city population Liberia 2025). In Maryland County, the Gbawiliken villages in the Barrobo Farjah District are home to the Grebo tribe. Residents here typically access the Ghee River as their water source. Maryland County has a population of 172 587 (city population Liberia 2025) and shares its borders with the Southwest of the Ivory Coast.

### Participant enrolment, FGS score card and urine sample collection

The study was based at a Ministry of Health outpost and made use of existing community outreach workers to make first contact with communities for study sensitization. Paper participant consent forms, with recruitment information sheets, were translated into the local language and lifestyle vernaculars dominant within each county with special attention on farming, fishing and business. These are common lifestyles associated with locally predominant economic activities and frequency of travel across neighbouring countries, which is a norm for many of the study participants. A cumulative target of enrolling a hundred women, aged 18–45, per county set the sampling frame, noting that initial over-recruitment was needed to acquire sufficient numbers. This varied locally by county, and many women were not sufficiently confident or empowered to report to a local health facility for gynaecological services.

Prior to enrolment, community health assistants (CHAs) fluent in local dialects explained the study, consent forms and procedures to participants. The study included completing an FGS score card, providing a urine sample and undergoing a gynaecological examination. Participants gave consent by signature or fingerprint, after which CHAs explained and assisted with the FGS score card, especially for illiterate participants. The FGS score card had 2 sections: Group 1, environmental and public health risk factors (bathing, swimming, swamp farming, fishing, fetching water, proximity <10 km to river and alternative water sources). A participant could accumulate a potential 36 points from this section. From Group 2, clinical symptoms/indicators (intermittent genital bleeding, post-coital bleeding, pelvic pain, haematuria, amenorrhea, abnormal discharge and painful urination), a participant could obtain a maximum 14 points. Each participant received a total FGS score out of 50, categorized as low < 25%, medium 25–49%, high 50–74% and very high ≥ 75%.

### Clinical and parasitological inspections

**Figure 2.**
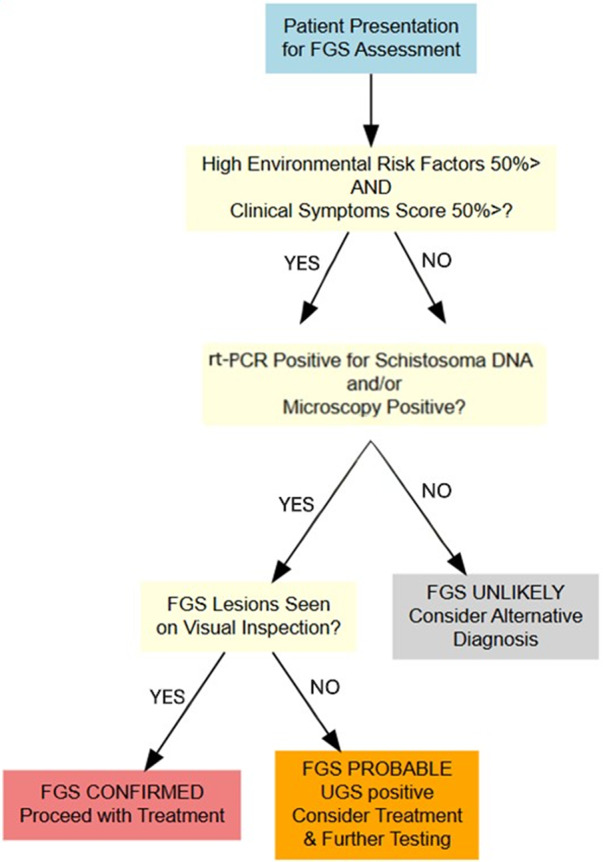
FGS/UGS decision tree.

Although a clinical and visual gynaecological exam was part of the initial consent form for the study signed by all participants, the CHAs assisted in obtaining additional verbal consent immediately before the clinical examination which took place in a private room. Each participant received a visual gynaecological examination by midwives who had been previously trained in clinical workshops by skilled and experienced gynaecologists at the Ministry of Health who had expertise in identifying FGS lesions. The gynaecological exam took no longer than 10 minutes. This involved the participant lying prone with insertion of a metal speculum with visual inspection alone of the participant’s cervico-vaginal surfaces (Figure 4). Referring to the WHO pocket atlas for lesions characteristic of FGS, this cursory inspection without a portable colposcope allowed the midwife to look for atypical characteristics of FGS lesions (grainy sandy patches, ulcers, homogeneous yellow sandy patches, rubbery papules, inflamed mucosa and contact bleeding) (COU**NTD**OWN FGS Intervention Manual 2018–2019). The midwives wrote the results from the visual inspection of each participant, noting a positive or negative if lesions were present or absent and the characteristics of FGS lesions seen. While UGS was confirmed in participants who had a positive microscopic or real-time polymerase chain reaction (rt-PCR) result for *Schistosoma* DNA, FGS was confirmed by participants who had UGS with high FGS scores (50% >), environmental risk factor and clinical symptom scores, in addition to typical FGS lesions seen upon visual inspection of cervico-vaginal surfaces (Figure 2).

Parasitological inspection of urine samples collected from each participant took place onsite. Approximately 30 mL of urine was collected from participants at healthcare centres between 10 am–2 pm and examined via microscopy for *S. haematobium* ova and other parasites. General urine analysis was initially conducted on the samples; samples were observed for appearance (colour, turbidity), and characteristics (presence of red or white blood cells and crystals). After general urine analysis, the samples were investigated for the presence of *S. haematobium* and other parasites via microscopy using the following method. The samples were mixed gently by inverting the urine container 2–3 times. A total of 14 mL was transferred to a 15 mL conical tube and centrifuged for 3–5 minutes at 1500–2000 rpm. The supernatant was removed with care taken not to disrupt the sediment. One drop of the sediment was placed on a glass slide and viewed under stereomicroscopy (Olympus CX33) at × 100 magnification. Results were recorded on urine analysis results sheets; the remaining sediment was then placed in a 2 mL tube containing 1 mL absolute ethanol for transfer to the UK and the Liverpool School of Tropical Medicine (LSTM) laboratory.

Total genomic DNA was subsequently extracted. Prior to real time (rt)-PCR analysis, as the samples had been stored in absolute ethanol, the samples were spun at 2000 rpm for 30 second and washed 3 times with distilled water to ensure that excess ethanol was removed prior to DNA extraction and rt-PCR screening. The DNA extracts were obtained by the (Qiagen) DNA mini blood and tissue kit following the instructions of the manufacturer, with the addition of a bead beating step. Prior to samples being incubated with ALT and Proteinase K buffer, each sample had a bead beating treatment where 0·45 g of 1·4 mm ceramic beads were added. Phocine Herpes Virus (PhHV) was used as a positive control for internal extraction. Negative extraction controls were also used for each round of DNA extraction.

### Generic and species-specific identification using high-resolution melt (HRM) RT-PCR analysis

Extracted DNA was then examined with a molecular DNA diagnostic real-time PCR assay reported by Cunningham et al. (2024) and Obeng et al. (2008) that enables *Schistosoma* identification and analysis. Two assays were used to first identify the presence of generic schistosome genome and then to determine the presence of any of the 6 schistosome species important in Africa. The first assay was a generic Schisto rt-PCR and PhHV assay, which is a singleplex assay. This verified the presence of schistosome nuclear DNA (nDNA) targeting a short ribosomal region of internal transcribed spacer 2 (ITS2). The second assay is a multiplex assay, which is a species-specific assay, mitochondrial DNA (mtDNA), high-resolution melt (HRM) rt-PCR assay. The multiplex assay targets the schistosome mitochondrial genome and various gene markers, these target markers vary in relation to schistosome species (Table 1). Both assays were run using a total volume of 12 µL. Within the reaction was 6 µL of the HRM Supermix (Qiagen), a concentration of 400 nM for each primer and 2 µL of template. Nuclease-free water was used to make up the remaining volume to 12 µL. The rt-PCR assays were run for 35 cycles to reduce background fluorescence, as previous studies showed that nonspecific products were produced when run for 40 cycles (Cunningham et al. 2018). Optimum annealing temperatures for nDNA HRM rt-PCR were 60 °C and 58 °C for mtDNA HRM rt-PCR.

**Table 1.** Primer list for species-specific mtDNA HRM rt-PCR assay

Target sp.	Primer name	Primer sequence	Primer Tm (°C)	Product size (bp)	Molecular target
*S. mansoni*	SchMnF	GGTTGAAGAGGAGGTTCGTG	60.0	121	tRNA-Lys
	SchMnR	GGTCGCAATATACTCGACACC	61.2		
*S. haematobium*	SchHmF2	GCTGTAAAGGTGGCTGATAGTAGC	65.0	126	tRNA-Lys
	SchHmR2	TATCAACTTAACTATGCACCTAGTG	60.0		
*S. margrebowiei*	SchMrgF2	GGATCACGAAGTTGGGCTATAC	62.0	78	ND4
	SchMrgR2	GAATATCAGCACAGACAATACTTGAAC	63.0		
*S. bovis*	SchBovF	CAACATAAGATGATTGTAGTTAGC	58.0	156	tRNA-Lys
	SchBovR	CTTTATTACTCGGCCACGATATG	60.9		
*S. curassoni*	SchCurrF	GTCGTGCTTTTGGTGATTAGC	59.0	120	tRNA-Lys
	SchCurrR	CCTACGCCCGATAAACTAAAC	59.0		
*S. mattheei*	SchMattF	GTTGGTTTCGTATTTTTTTATGTTAAGG	61.3	74	ND6
	SchMattR	CTAACTTAGCGCTTCACAAAATGC	62.0		

### Data analysis

Raw data were compiled in Excel and analyzed in R (v4.0.2). UGS prevalence differences across sites were tested using chi-squared tests for proportions; when < 5, Fisher’s exact test was used. The null hypothesis was equal prevalence across sites; *P*<0·05 indicated significance. Pairwise site comparisons used Fisher’s exact tests with Bonferroni-adjusted *P*-values. FGS Score Card Group 1 and Group 2 results were compared with parasitology and clinical findings to assess UGS/FGS. Multivariate logistic regression modelled associations between environmental/public health risk factors and UGS (Figure 5). Data from participant’s visual gynaecological exam was analyzed against their UGS results to determine participants who had FGS. Exact (Clopper–Pearson) 95% confidence intervals were computed per site and for overall FGS prevalence.

## Results

The basic demographic characteristics for study participants are shown in Table 2. Despite best efforts to recruit without bias, Lofa County participants were predominantly older while Nimba County participants were younger. Most women across the 4 counties listed farming as their main occupation whilst many women listed fishing as their main occupation. Of note, most women had no formal education with 31·5% having attended elementary school education alone, with just over 1·5% having received secondary school education.

**Table 2.** Key demographical characteristics of participants by county

County	Average age of participants	Occupation		Level of education	
Lofa	35	Farmers	75·0%	Elementary school	70·0%
		Fisher women	5·0%		
		Business women	20·0 %	Secondary school	30·0%
Bong	25	Farmers	94·0%	Elementary school	45·0%
		Fisher women	6·0%	Secondary School	6·0%
Nimba	23	Farmers	90·0%	Elementary School	60·0%
		Fisher women	10·0%	Secondary School	40·0%
Maryland	30	Farmers	93·0%	Elementary school	5·0%
		Fisher women	7·0%	Secondary school	0·0%

### UGS positives and molecular analysis

A total of 22 women (5·5%) out of 400 women across all 4 sites tested positive for generic schistosome species after HRM rt-PCR analysis (Table 3). This meant that 22 women had UGS. Nimba had the highest UGS prevalence rate at 11% for 100 participants followed by Maryland county which had a rate of 6% for 100 participants. Bong County had a 4% prevalence rate, while Lofa had just 1% UGS prevalence from 100 participants. Only 1 of the samples showed a positive result for *S. mansoni* after species-specific analysis from the mtDNA HRM rt-PCR multiplex assay. This was from a participant in Nimba County. All other samples were found positive for *S. haematobium* showing that 99% of UGS positive cases from the study had *S. haematobium* as the causative agent. Of interest here, *S. mansoni* was the only other schistosome species found amongst the samples. There was no evidence of hybridization from the HRM rt-PCR analysis.

**Table 3.** UGS prevalence % for women aged (18–45) in Liberia

Study site	N	No. positive generic for Schistosome DNA	*Schistosoma* spp. prevalence (%) 95% CI
Nimba	100	11	11.0 (5·6−18·8)
Maryland	100	6	6.0 (2·2−12·6)
Bong	100	4	4.0 (1·1−10·0)
Lofa	100	1	1.0 (0·0−5·5)
**Total examined**	**400**	**22**	**5.5 (3·4–8·2)**

### FGS score cards and FGS risk factors

**Figure 3.**
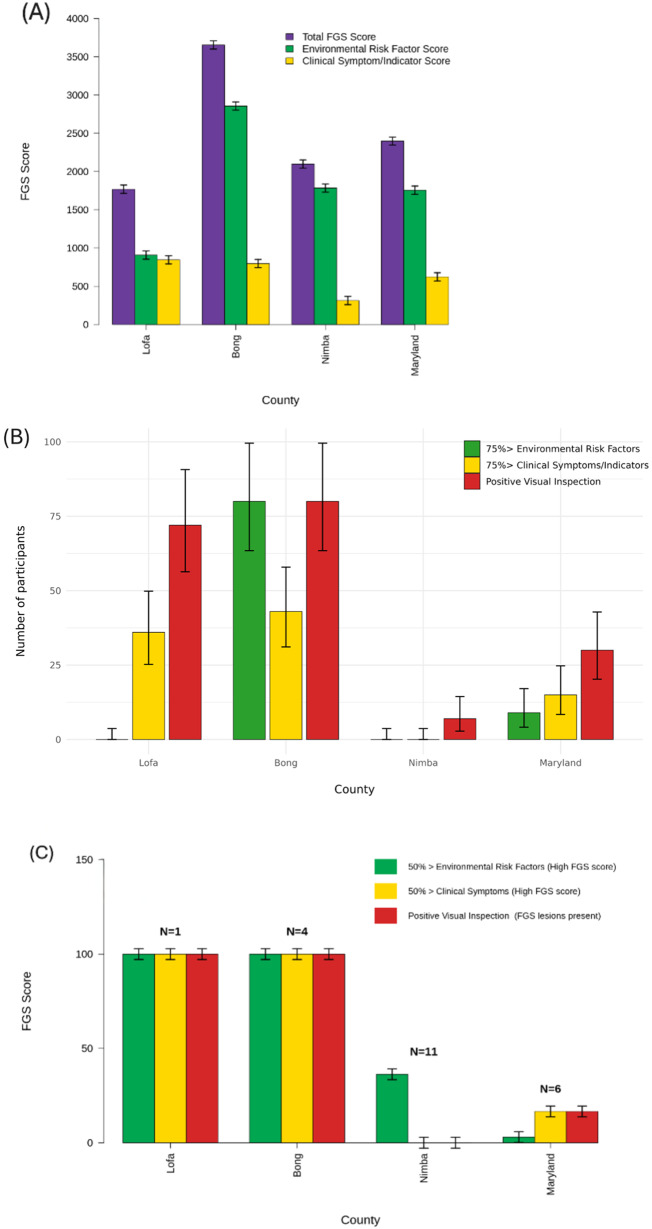
**(A)** Total FGS score per county, total environmental risk factors score, total clinical symptoms score and total number of participants positive for FGS lesions during visual inspection (grainy sandy patches, ulcers, homogeneous yellow sandy patches, rubbery papules, inflamed mucosa, contact bleeding) for 100 participants in each county. **(B)** Number of women with very high FGS scores (75% >) for environmental risk factors,75% > clinical indicators and women positive for FGS lesions per county. **(C)** Shows the percentage of participants positive for UGS with a high FGS score (50% >) for environmental risk factors, 50% > FGS clinical indicators and the percentage of UGS positive participants who had FGS lesions.

FGS score cards from 400 participants (100 per county) were analyzed to identify environmental and clinical risk patterns and compare FGS risk by location. There were notable county-level differences (Figure 3A). Although Nimba had the most UGS-positive cases, its total FGS score (2099/5000) ranked third. Bong County had the highest overall FGS score, followed by Maryland, Nimba and Lofa. Environmental risk factor scores were as follow: Bong (2857), Nimba (1785), Maryland (1757), Lofa (908). Lofa had the highest clinical symptom scores per county (846), followed by Bong (798), Maryland (642) and Nimba (314). The share of women with very high scores (≥75%) also differed (Figure 3B). Overall, Bong County showed the greatest combined FGS risk with 80% of participants in the very high FGS risk category.

**Figure 4.**
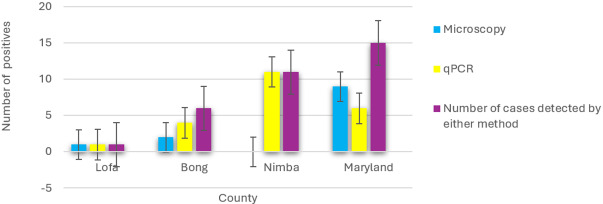
**(A)** Shows the midwife examining the cervix of a participant for possible FGS lesions. **(B)** The speculum used for examination. **(C)** Illustrates the healthy cervix of a participant with no typical FGS lesions. **(D)** Shows the cervix of a participant with FGS lesions, yellow sandy patches and genital ulceration. **(E)** The front of Foya Health Centre in Lofa County, the site the above examination took place.

### Gynaecological examination (visual inspection and FGS scores)

During visual inspection, typical FGS lesions (grainy sandy patches, ulcers, homogeneous yellow sandy patches, rubbery papules, inflamed mucosa, contact bleeding) were noted among 100 participants per county. Bong County had the highest number of participants with FGS lesions, 80/100. Lofa 72/100, Maryland 30/100, Nimba had the least participants with FGS lesions 9/100 participants. Grainy sandy patches were the most common lesion found, followed by homogeneous yellow sandy patches; ulcers and rubbery papules were least common (Table 4). FGS confirmation was based on positive RT-PCR for *Schistosoma* DNA (UGS), a high FGS score (>50%) for environmental risk factors and clinical symptoms (Figure 3C) and typical FGS lesions seen on visual inspection. All 4 UGS-positive participants from Bong County met all criteria (100%). Lofa had 1 UGS-positive participant who also confirmed FGS. Nimba had the highest UGS rate but no confirmed FGS (low FGS scores and no lesions). Maryland had a single participant who met the criteria for FGS. Overall, Bong County had the highest confirmed FGS prevalence, while Nimba had none despite high UGS positivity (Table 5).

**Table 4.** Category of FGS lesions seen on gynaecological visual inspection of each participant, 400 participants 100 participants per county

County	*N*	Grainy sandy patches	Ulcers	Homogeneous yellow sandy patches	Rubbery papules	Inflamed mucosa	Contact bleeding
Lofa	72	25	4	18	4	11	10
Bong	80	28	4	20	4	12	12
Nimba	7	3	0	2	0	1	1
Maryland	30	11	1	7	1	5	5

**Table 5.** FGS prevalence % for women aged (18–45) in Liberia

Study site	N	No. positive for FGS (UGS positive and FGS lesions present)	FGS prevalence (%) 95% CI
Nimba	100	0	0.0 (0–3·62)
Maryland	100	1	1.0 (0·03–5·45)
Bong	100	4	4.0 (1·1–9·93)
Lofa	100	1	1.0 (0·03–5·45)
Total examined	400	6	1·5 (0·55–3·24)

### UGS and FGS risk factors

**Figure 5.**
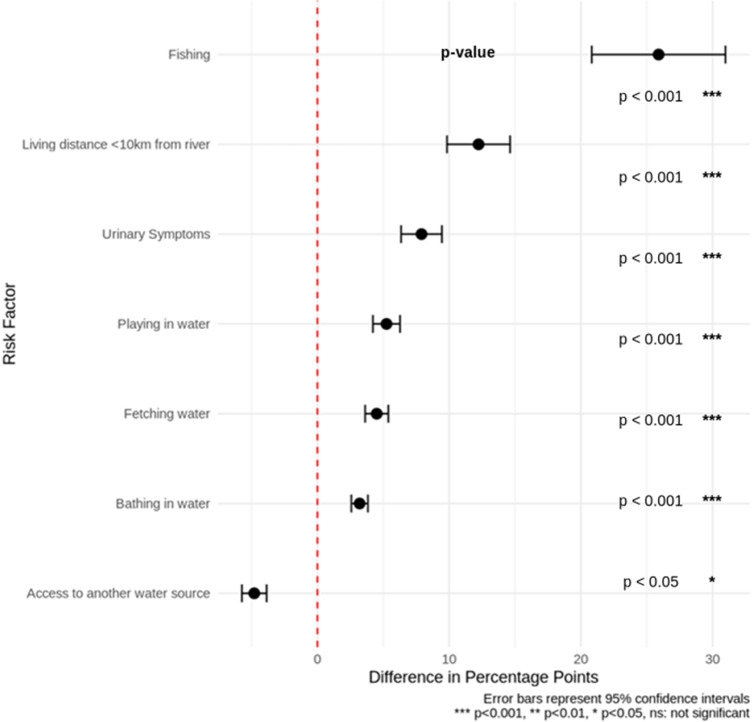
Shows the difference in percentage points of risk factors for (DNA ±) participants. The dotted red line at 0 indicates no difference between groups for each risk factor. Data points to the right of the line (positive values) are associated with a higher difference in percentage points (i.e. increased risk compared to the reference group). Data points to the left of the line (negative values) indicate a lower difference (i.e. decreased risk). All risk factors shown have positive differences, meaning they are associated with an increased risk of UGS and, as a result, an increased risk of FGS. Error bars represent 95% confidence intervals for 400 participants (100 participants per county).

Seven categories showed clear percentage differences between *Schistosoma* DNA-positive and DNA-negative participants (Figure 5). Participants engaging in certain water-related activities were more likely to test positive for the schistosome genome. Group 1 categories which were significant environmental factors, were playing in water, bathing, fishing, fetching water and living < 10 km from a river all were (*P*<0·001), while access to another water source was (*P*<0·05). Urinary symptoms such as haematuria, painful urination and leaking (*P*<0·001) were the significant clinical symptoms from Group 2. Except for those with access to alternative water sources, *Schistosoma* DNA-positive participants were higher across all categories. Overall, infection risk increased with frequent river contact, while access to other clean water sources reduced UGS and potential FGS risk.

### Microscopic sensitivity and UGS diagnosis

**Figure 6.**
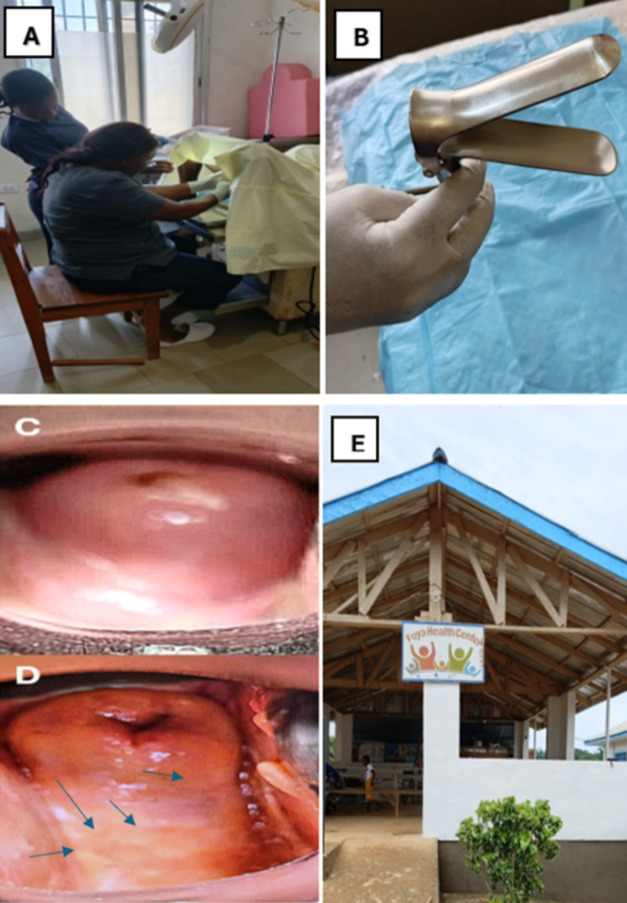
Number of *Schistosoma* *haematobium* cases per county, detected by urine-based microscopy and/or rt-PCR.

The detection of schistosome eggs by microscopy was compared with results from HRM rt-PCR analysis to look at the sensitivity of microscopic analysis for UGS detection (Figure 6). About 54% of the urine samples, which were positive for *Schistosoma* DNA, were also positive via microscopy. The total number of cases detected by microscopy or rt-PCR was for Lofa, 1 case, Bong County, 6 cases, Nimba, 11 cases and Maryland 15 cases. Although urine microscopy identified 9 cases with schistosome ova in Maryland, only 6 samples were confirmed positive by DNA testing. In Bong County, 4 samples were positive for *Schistosoma* DNA, with microscopy detecting 50% of these. Nimba County recorded the highest number of *Schistosoma* DNA-positive results (11), but none were detected by microscopy. In contrast, Lofa had a single *Schistosoma* DNA-positive sample, which microscopy also identified. There was no evidence of other parasites present.

## Discussion

As this study is the first formative appraisal for FGS score card results with clinical, molecular and parasitology evidence, it helps to close the research gap in FGS and develop a better ameliorative response in Liberia with provisional insights on the trend of FGS in Liberia. We also focus on risk factors that may be strongly associated with presenting with FGS in each county locally. There were limitations, however, that should be considered. Foremost, colposcopy is not available within primary care settings in Liberia, and only visual inspection with a speculum is possible. It was important for the purpose of this study to probe methodology which could be easily reproduced in resource-poor settings where FGS is endemic. Our relatively small sample size in the positive group (*n* = 22) led to much wider confidence intervals (approximately ± 20%) compared to the negative group (*n* = 376). Nonetheless, the most reliable differences were seen in fishing activities and in living distance(s) from a river. In general, further research with larger sample sizes would be beneficial to confirm findings from the study and help spatially target those women most at risk.

### Demographical information

Analysis showed a link between occupation and UGS prevalence across the 4 counties. Nimba, with the highest UGS rate (11.0%), also had the most participants engaged in fishing (10.0%). This was followed by Maryland (6.0% UGS, 7.0% fishing), Bong (4.0% UGS, 6.0% fishing) and Lofa (1.0% UGS, 5.0% fishing). Overall, Nimba had 50.0% more women who listed fishing as their occupation than Lofa, supporting fishing as a key exposure activity. No clear correlation was found between age and UGS positivity, though Nimba’s UGS-positive group had the youngest average age (23 years). Education levels varied widely. About 100% of participants from Nimba and Lofa had received a formal education. Nimba had 40.0% of participants with secondary schooling, although Bong County had 51.0% who had formal education, only 6.0% reached the secondary level. Only 5.0% of Maryland’s participants had elementary education; none received a secondary school education. There was no association between education level and UGS positivity across counties.

### Observations from FGS score cards

With an environmental risk factor score of 1785, Nimba County, which had the highest UGS positivity, scored 50% higher than Lofa, the county with the fewest UGS cases. Overall, 99% of UGS-positive participants across all counties had high environmental risk factor scores (>50%). Counties with more UGS-positive cases, such as Bong and Nimba, also had higher total environmental risk factor scores than Maryland and Lofa (Figure 3A). However, high environmental risk factor scores did not always correspond with high clinical symptom scores. Some UGS-positive women showed few clinical symptoms, while some UGS-negative participants reported many. This indicates that environmental exposure may be a stronger predictor of UGS and potential FGS risk than clinical symptoms, which can overlap with other conditions such as STIs.

### Clinical examination of FGS

Although Nimba recorded the most UGS positive participants, it showed the lowest presence of visible FGS lesions on examination and no cases that met the study’s confirmation criteria; notably, none of the UGS positive women there had visible lesions. In contrast, Bong exhibited the greatest burden of visible lesions, and all women with UGS in that county also had lesions on inspection. Lofa, despite having very few UGS positive cases, showed a high prevalence of visible lesions overall, while Maryland displayed a modest presence of typical lesions, including among at least one UGS positive participant. Collectively, these patterns indicate that visible genital lesions alone are not a reliable indicator of confirmed FGS, as they can occur in women who test negative and may reflect other gynaecological or infectious conditions.

### Prevalence of UGS and FGS

Confidence intervals for UGS prevalence were calculated using standard proportion formulas (Table 3). A Chi-square test showed statistically significant differences in prevalence between counties (χ^2^ = 9·636, *df* = 3, *P* = 0·021), confirming that UGS infection rates vary by location. A clear north–south gradient was observed, with Nimba County showing significantly higher prevalence. Although confidence intervals were wide due to the small number of positive cases, equal sample sizes across sites (*N* = 100 each) support reliable comparisons. Overall, UGS prevalence in Liberia was 5·5%, and FGS prevalence was 1·5%, both relatively low compared to other SSA countries. For instance, FGS prevalence among young women (11–20 years) has been reported at 57.2% in Zambia and 79·5% in Ghana (Mberu et al. 2025), while a meta-analysis across 21 SSA countries found an average of 17·5% (Shams et al. 2022). Differences in methodology and participant age may explain Liberia’s lower rates, as the study sample had an average age of 29 years. Targeting younger age groups may reveal higher prevalence levels. To prevent the escalation of FGS in these vulnerable regions, the Liberian Ministry of Health should collaborate with stakeholders to strengthen Water, sanitation and hygiene (WASH) initiatives in schistosomiasis-endemic rural regions, inclusive of appropriate menstrual health management perspectives (Stothard, Odiere and Phillips-Howard, 2020), particularly as these communities share their borders with countries which have high *S. haematobium* prevalence rates (Hodges et al. 2011).

### Parasitological examinations

Although Lofa’s microscopic result showed that the microscopist had 100% accuracy in their analysis, as they detected the county’s only positive sample and accurately noted the rest as negative, overall, across the counties microscopic analysis only accurately captured 40·9% of samples found positive for *S. haematobium* molecular diagnosis (Figure 6). This reflects the challenges faced in a resource-poor setting trying to obtain adequate healthcare delivery post-war. Several factors may contribute to such results. Possible factors include a lack of human resource development of lab technicians in critical areas such as schistosome detection, possible poor quality of microscopes and other laboratory equipment. However, Meurs et al. (2015) conducted a study in Senegal and Kenya where an in-depth evaluation of ITS2 PCR was compared with standard microscopy for *Schistosoma* detection. Their study showed that ITS2 PCR was more sensitive than standard microscopy. This indicates that DNA analysis by rt-PCR would have detected samples missed by microscopical analysis even in more developed settings.

### Risk factors

Participants positive by urinary PCR generally had higher percentages across most environmental risk factors and clinical symptoms. The most common risk factor for both groups was living within 10 km of a river, while fishing showed the largest difference between positive and negative participants (Figure 5). Access to another water source was the only factor more common in PCR-negative participants. Confidence intervals (95%) were calculated for each proportion using standard error formulas, given sample sizes of 22 positive and 376 negative participants. Fishing, positive 77·2% (CI: 59·7–94·7%), negative 51·3% (CI: 46·2–56·4%) non-overlapping CIs indicate a statistically significant risk factor, likely due to longer river exposure during fishing. Living < 10 km from a river, positive 81·8% (CI: 65·7–97·9%), negative 69·6% (CI: 64·9–74·2%) marginally overlapping CIs suggest a possible but not definitive risk.

Bathing in river water, positive 59·0% (CI: 38·4–79·6%), negative 55·8% (CI: 50·8–60·8%), overlapping CIs indicate no significant difference. Urinary symptoms, positive 31·8%, negative 23·9%, had wide CIs due to small sample size, difference is not statistically significant. Overall, fishing and proximity to rivers appear as the strongest environmental risk factors for UGS and potential FGS, while bathing and urinary symptoms were less predictive.

## Conclusion

From the evidence presented here, the prevalence of FGS in Liberia appears to be low compared to other SSA countries, largely restricted here to women living in areas where there are high environmental risk factors. As environmental risk factors showed a statistically significant difference in UGS and potentially FGS incriminations, the Ministry of Health and its partners should explore avenues which may reduce women being exposure to these environmental risk factors. With focus placed on counties in particular, Bong, Nimba and Maryland, which had high FGS environmental risk factor total scores. As fishing activities showed the most significant difference amongst all categories, UGS and FGS should be considered as potential occupational hazards for women who rely on fishing for their livelihood. Overall, the FGS score card is a useful tool in the diagnostic process for FGS by identifying specific women who are high risk for FGS, but it may overestimate when compared with clinical and parasitological methods.

## Data Availability

Data will be made available on request.
